# Altered Excitation–Inhibition Balance and mGluR_1/5_-Driven Plasticity in the Motor Cortical Surface in a Rat Model of Parkinson’s Disease

**DOI:** 10.3390/ijms27177564

**Published:** 2026-08-24

**Authors:** Hongseong Shin, Yoon Ji Kwon, Hyunjung Hwang, Taewoo Ko, Eun Bi Choi, Yang Tae Kim, Yu Mi Han, Jae Geun Kim, Qiang Zhou, Sungchil Yang, Sunggu Yang

**Affiliations:** 1 Department of Nanobioengineering, Incheon National University, Incheon 22012, Republic of Korea; hong48078@gmail.com (H.S.); ughssun@gmail.com (H.H.);; 2 Center for Brain-Machine Interface, Incheon National University, Incheon 22012, Republic of Korea; jgkim@inu.ac.kr; 3 Department of Neuroscience, City University of Hong Kong, 83 Tat Chee Avenue, Kowloon, Hong Kong SAR, China; yjkwon3-c@my.cityu.edu.hk; 4 Division of Life Sciences, College of Life Sciences and Bioengineering, Incheon National University, Incheon 22012, Republic of Korea; dmsql6229@inu.ac.kr (E.B.C.); rabit7890@gmail.com (Y.T.K.); 5 Research Institute of Medical Science, Sungkyunkwan University School of Medicine, Seoul 16419, Republic of Korea; prb68@naver.com; 6 State Key Laboratory of Chemical Oncogenomics, Guangdong Provincial Key Laboratory of Chemical Genomics, Peking University Shenzhen Graduate School, Shenzhen 518055, China; zhouqiang@pkusz.edu.cn; 7 gBrain Inc., Incheon 21984, Republic of Korea

**Keywords:** Parkinson’s disease, motor cortex, synaptic plasticity, metabotropic glutamate receptor, sensorimotor deficits

## Abstract

Parkinson’s disease (PD) is characterized by progressive dopaminergic degeneration and maladaptive motor cortical plasticity. However, the cellular pathways underlying cortical surface activity in the primary motor cortex (M1) remain unclear, despite serving as a potential target for electrotherapy. We investigated the excitatory–inhibitory (E-I) balance and synaptic plasticity of superficial M1 circuits in a unilateral 6-hydroxydopamine (6-OHDA)-induced rat model of PD. Using extracellular local field potential and whole-cell patch recordings from the contralateral and ipsilateral M1 hemispheres of hemi-parkinsonian rats, we observed a significantly elevated field excitatory postsynaptic potential (fEPSP) input–output function but unchanged intrinsic neuronal excitability in the M1 superficial layer. An altered relative contribution between alpha-amino-3-hydroxy-5-methyl-4-isoxazolepropionic acid receptor (AMPAR)- and N-methyl-D-aspartate receptor (NMDAR)-mediated transmission was reflected by a significantly increased AMPA/NMDA ratio. Markedly reduced inhibitory synaptic tone was also evidenced by the decreased amplitude and frequency of spontaneous inhibitory postsynaptic currents (sIPSCs), supporting an E-I imbalance favoring excitation in PD. Furthermore, group I metabotropic glutamate receptor (mGluR_1/5_)-dependent long-term depression (LTD) was abolished in the ipsilateral PD hemisphere, whereas NMDAR-dependent LTD remained intact. In summary, dopamine depletion appears to enhance network excitation and disrupt mGluR_1/5_-mediated control of M1 surface circuitry. Our findings identify altered cortical surface mGluR-dependent plasticity in the hemi-parkinsonian model; however, the relationship between these electrophysiological alterations and individual motor outcomes remains to be determined.

## 1. Introduction

Parkinson’s disease (PD) is a progressive neurodegenerative disorder characterised by the loss of dopaminergic neurons, primarily in the substantia nigra *pars compacta* (SNc), leading to profound disruptions in motor function [1,2,3]. A major consequence of this nigral degeneration is the weakening of motor command and control, to and from the primary motor cortex (M1). The M1 critically executes precise motor commands; therefore, disruptions in this area are directly responsible for the motor deficits observed with PD [4,5]. In particular, sensorimotor cortex surface layers are thought to determine cortical map organisation, influence associative information processing, and play a role in learning [6,7,8]. Although the motor cortical surface is less understood, identifying pathological signatures in this region may provide novel targets for therapeutic interventions against PD as cortical surface plasticity is being increasingly recognized as a key factor in diverse neurological disorders [7,9,10,11,12]. Furthermore, surface recording and stimulation via electrocorticography can provide an electrotherapeutic platform for the effective modulation of M1 cortical surface activity [13,14,15,16].

Several previous studies have reported altered synaptic plasticity in the M1 in PD [4,5,17]. Both long-term potentiation (LTP)- and long-term depression (LTD)-like plasticity are impaired in patients with PD [18]. Metabotropic glutamate receptors (mGluRs) are key modulators of synaptic plasticity in sensorimotor cortices, and growing evidence indicates that dysfunction of these receptors contributes to PD-related motor impairments [19,20]. Group I mGluRs, which encompass mGluR_1_ and mGluR_5_, not only influence dopaminergic signaling in the healthy basal ganglia but also are found to be involved in the progression of dopaminergic degeneration in an animal model of PD [21]. Selective blockade of mGluR_5_ in the M1 reduces spontaneous locomotion and motor coordination [22]. As group I mGluRs are expressed in most sensorimotor neurons across various animal species [23,24,25], these findings strongly suggest a crucial role of mGluRs in motor function. Previously, we also demonstrated enhanced mGluR_1/5_-driven LTP at the M1 surface in an animal model of PD, and provided transcriptomic evidence indicating that genes associated with mGluR-dependent LTP, such as mGluR_5_, dopamine receptor 5, and G protein-mediated intracellular molecules, are altered in the M1 surface in the context of PD pathology [11]. However, it remains unknown whether changes in mGluR-mediated LTD are also associated with PD.

In this study, we aimed to investigate the roles of mGluRs in PD pathology at the M1 cortical surface in a rat model of 6-hydroxydopamine (6-OHDA)-induced PD. We found a reduction in mGluR_1/5_-driven synaptic depression, concurrent with an increase in the alpha-amino-3-hydroxy-5-methyl-4-isoxazolepropionic acid receptor (AMPAR)/N-methyl-D-aspartate receptor (NMDAR) ratio, and a decrease in inhibitory postsynaptic currents. Our findings identify altered cortical surface mGluR-dependent plasticity in the hemi-parkinsonian model; however, the relationship between these electrophysiological alterations and individual motor outcomes remains to be determined.

## 2. Results

### 2.1. Behavioural Validation of the Hemi-Parkinsonian Model

**Figure 1:**
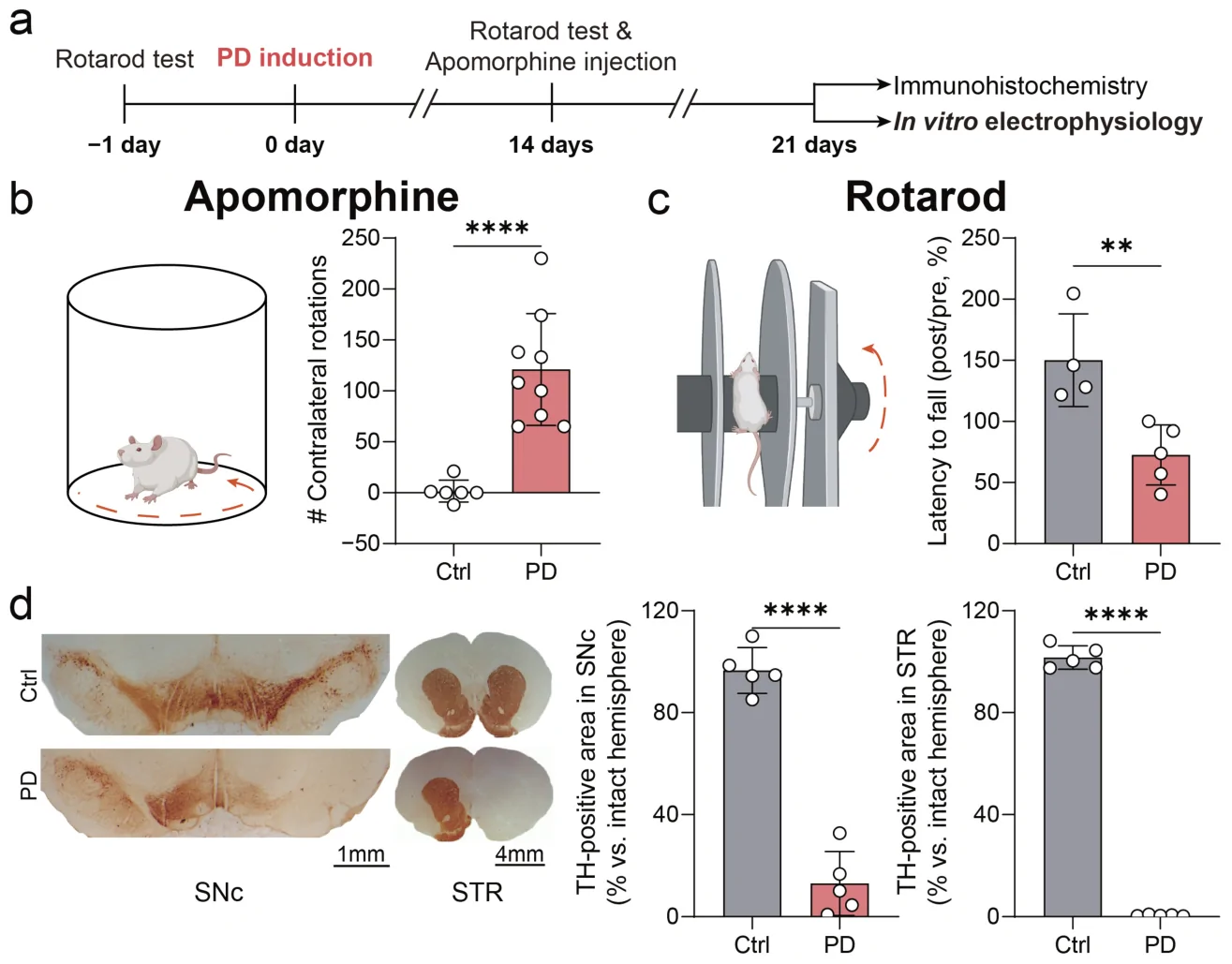
Experimental validation of the 6-hydroxydopamine (6-OHDA)-induced hemi-parkinsonian model. (**a**) Experimental timeline. (**b**) Confirmation of dopaminergic lesions by contralateral rotations in 6-OHDA-lesioned rats following apomorphine administration (0.5 mg/kg, subcutaneous). (**c**) Comparison of latency to fall from the rod between the control (Ctrl) and Parkinson’s disease model (PD) groups. (**d**) Tyrosine hydroxylase (TH) immunostaining of the substantia nigra pars compacta (SNc) and striatum (STR), showing substantial dopaminergic neuron loss in PD rats. Gray indicates the Ctrl group, and red indicates the PD group. All data are presented as means ± standard deviation. ** *p* < 0.01, **** *p* < 0.001.

PD model animals were generated by unilaterally administering 6-OHDA to the medial forebrain bundle of rats. Two weeks later, the rats were subjected to apomorphine-induced rotation and rotarod tests to confirm the behavioral symptoms of PD (Figure 1a). In the apomorphine-induced rotation test, PD rats showed significantly increased rotating behavior compared to control (Ctrl) rats, which exhibited minimal rotational behavior (PD: 121.00 ± 54.81, *n* = 9 vs. Ctrl: 1.67 ± 10.67, *n* = 6; *t*(8.890) = −6.354, *p* < 0.001, Hedges’ *g* = 2.582, two-sided independent-sample *t*-test; Figure 1b). The rotarod test also revealed significant motor impairments in PD rats. After normalizing data to the pre-test level, the latency to fall off the rotarod was decreased in the PD group but increased in the Ctrl group (PD: 72.68% ± 24.60%, *n* = 5 vs. Ctrl: 150.07% ± 37.88%, *n* = 4; *t*(7) = 3.722, *p* = 0.007, Hedges’ *g* = −2.219, two-sided independent-sample *t*-test; Figure 1c). These behavioral observations demonstrate successful establishment of the PD model.

One week after behavioral tests, tyrosine hydroxylase (TH) immunostaining was performed to additionally confirm the loss of dopaminergic neurons. In PD rats, the TH-positive area in the 6-OHDA-injected hemisphere (normalized to the untreated hemisphere) decreased significantly in both the SNc and striatum (STR) (PD_SNc_: 13.02% ± 12.53%, *n* = 5 vs. Ctrl_SNc_: 96.58% ± 9.01%, *n* = 5; *t*(8) = 12.107, *p* < 0.001, Hedges’ *g* = −6.916, two-sided independent-sample *t*-test; PD_STR_: 0.53% ± 0.29%, *n* = 5 vs. Ctrl_STR_: 101.63% ± 4.57%, *n* = 5; *t*(4.031) = 49.344, *p* < 0.001, Hedges’ *g* = −28.202, two-sided independent-sample *t*-test; Figure 1d). These results confirm that dopaminergic lesions caused motor defects in our PD rat model.

### 2.2. Increased Synaptic Excitability, but Not Intrinsic Excitability, of the M1 Cortical Surface Associated with PD Pathology

**Figure 2:**
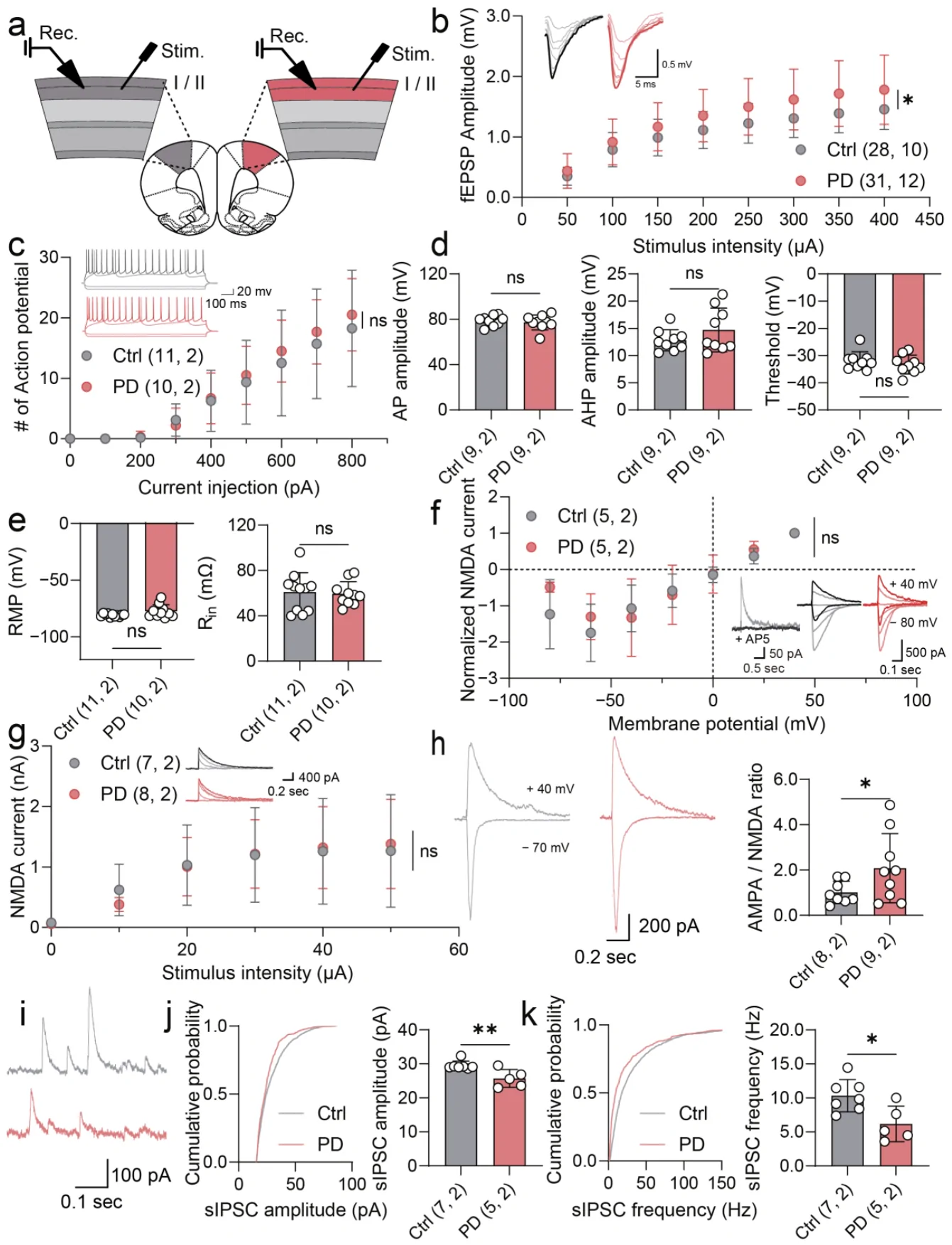
Electrophysiological characterization of synaptic and intrinsic properties in the M1 superficial layer of Parkinson’s disease (PD) model rats. (**a**) Schematic diagram of a coronal M1 slice showing the placement of a concentric bipolar stimulating electrode and a glass recording electrode within the superficial layers for extracellular field recordings, and a glass pipette targeting superficial-layer pyramidal neurons for voltage- and current-clamp experiments. (**b**) Field excitatory postsynaptic potential (fEPSP) input–output curve. fEPSPs were evoked in the superficial layer with single pulses (0.1 Hz, 100 µs), and the peak-to-baseline amplitude was measured (0–400 µA in 50 µA steps; modified from our previous work [11]). (**c**) Action potential (AP) firing rate. Intrinsic excitability was quantified in current clamping by biasing neurons to −70 mV and injecting depolarizing steps from 0 to +800 pA in 100 pA increments. The number of APs per step increased with the current in both groups, without a group effect. Representative traces showing membrane potential responses to step current injections (−400, −200, +300, and +600 pA). (**d**) AP intrinsic properties. From the first suprathreshold spike, AP amplitude (threshold-to-peak), afterhyperpolarization (AHP) amplitude (threshold-to-most-negative deflection), and AP threshold (dV/dt > 20 mV/ms) were extracted. These parameters were comparable between control (Ctrl) and Parkinson’s disease model (PD) neurons, indicating preserved spike generation mechanisms. (**e**) Membrane intrinsic properties. Resting membrane potential (RMP) was measured 3 min after break-in; input resistance (R_in_) was computed from the voltage deflection to a −100 pA step. (**f**) Current-voltage (I-V) relationship of evoked N-methyl-D-aspartate receptor (NMDAR) currents. NMDA EPSCs were pharmacologically isolated at +40 mV in the presence of NBQX (10 µM; alpha-amino-3-hydroxy-5-methyl-4-isoxazolepropionic acid receptor [AMPAR] antagonist) and gabazine (10 µM; GABA_A_R antagonist); AP5 (100 µM; NMDAR antagonist) abolished the residual current, confirming the isolation of NMDA EPSCs. The holding potential was stepped from +40 to −80 mV to construct I-V curves. (**g**) Input–output relationship of evoked NMDAR currents. Under the same pharmacological treatment as (**f**), the stimulation intensity was increased from 0 to 50 µA (10 µA increments). The peak NMDA current scaled with the stimulus intensity and did not differ between groups. (**h**) AMPA/NMDA ratio. AMPAR EPSCs were recorded at −70 mV, and NMDAR EPSCs were recorded at +40 mV from the same cell (gabazine present). The AMPA/NMDA amplitude ratio was elevated in PD neurons, indicating an altered relative contribution of AMPAR- and NMDAR-mediated transmission. (**i**–**k**) Spontaneous inhibitory postsynaptic currents (sIPSCs) were recorded at 0 mV in the presence of NBQX (10 μM) and AP5 (100 μM) to pharmacologically isolate inhibitory synaptic events. Cumulative probability plots and summary graphs show a reduced sIPSC amplitude and frequency in PD neurons compared with controls. Gray indicates the Ctrl group, and red indicates the PD group. All data are presented as means ± standard deviation. * *p* < 0.05, ** *p* < 0.01.

Altered synaptic transmission and plasticity in motor cortical circuits are well established in PD and lead to abnormal control of motor functions [26,27]. However, the specific contribution of the M1 surface layer to PD remains poorly understood. Accordingly, we investigated whether PD alters neuronal activity at the cortical surface. As in our previous study [11], we performed local field potential and whole-cell patch-clamp recordings of layer 2 pyramidal neurons in hemi-parkinsonian PD model rats (Figure 2a). A significantly increased field excitatory postsynaptic potential (fEPSP) input–output function (i.e., synaptic excitability) of the M1 cortical surface was observed in the ipsilateral PD hemisphere compared with the contralateral Ctrl hemisphere (Figure 2b, Table 1). Hence, we aimed to characterize the cellular pathways behind the enhanced synaptic responses to electrical stimulation in the M1 cortical surface. The fEPSP input–output data shown in Figure 2b were previously reported in our previous work and are reproduced here with a modified graphical presentation for comparison with the present cellular electrophysiological findings.

We first tested whether the intrinsic excitability of individual pyramidal neurons would be altered in response to long current injections. We quantified neuronal excitability as the number of action potentials (AP) generated in response to depolarizing current steps (0 to +800 pA, 100 pA increments). Both Ctrl and PD neurons displayed a gradual increase in the firing frequency with increasing current injections, with no detectable difference between the groups (*F*(8, 149.332) = 1.091, *p* = 0.373, repeated-measures linear mixed-effects model; Figure 2c, Table 2). Next, we analyzed various parameters of AP and intrinsic membrane properties. The AP amplitude, afterhyperpolarization (AHP) amplitude, and threshold voltage did not significantly differ between the groups (AP amplitude, PD_amplitude_: 77.23 ± 6.61, *n* = 9 vs. Ctrl_amplitude_: 78.81 ± 4.57, *n* = 9; *F*(1, 16) = 0.346, *p* = 0.564, Hedges’ *g* = −0.265; AHP amplitude, PD_AHP_: 14.71 ± 4.01, *n* = 9 vs. Ctrl_AHP_: 12.81 ± 1.95, *n* = 9; *F*(1, 15.711) = 2.124, *p* = 0.165, Hedges’ *g* = 0.574; Threshold, PD_threshold_: −33.29 ± 3.48, *n* = 9 vs. Ctrl_threshold_: −31.90 ± 3.34, *n* = 9; *F*(1, 16) = 0.746, *p* = 0.400, Hedges’ *g* = −0.388, linear mixed-effects model; Figure 2d). Similarly, neither the resting membrane potential (RMP), measured 3 min after break-in, nor the input resistance (R_in_), which was assessed at a hyperpolarizing current injection of −100 pA, differed significantly between the groups (RMP, PD: −77.35 ± 5.78, *n* = 10 vs. Ctrl: −80.82 ± 1.29, *n* = 11; *F*(1, 18.115) = 3.560, *p* = 0.075, Hedges’ *g* = 0.815; R_in_, PD: 59.46 ± 10.66, *n* = 10 vs. Ctrl: 60.86 ± 17.14, *n* = 11; *F*(1, 19) = 0.049, *p* = 0.828, Hedges’ *g* = −0.093, linear mixed-effects model; Figure 2e). These data show that PD pathology did not alter the AP and membrane properties that determine the intrinsic excitability of M1 surface neurons, indicating that intrinsic membrane properties are not associated with the enhanced synaptic excitability observed in PD.

Given the lack of involvement of intrinsic excitability and presynaptic transmission [11], we then questioned whether the increased synaptic excitability would be associated with postsynaptic glutamatergic receptors. We therefore examined the roles of AMPARs and NMDARs in M1 surface layer pyramidal neurons. NMDAR-mediated excitatory postsynaptic currents were recorded at a holding potential of +40 mV in the presence of 2,3-dioxo-6-nitro-7-sulfamoyl-benzo[f]quinoxaline (NBQX, 10 μM; AMPAR antagonist) and gabazine (10 μM; GABA_A_ receptor antagonist). Treatment with (2R)-amino-5-phosphonovaleric acid (AP5, 100 μM; NMDAR antagonist) completely abolished the residual current, confirming the pharmacological isolation of NMDAR-mediated responses. For NMDA EPSCs, the current–voltage (I-V) relationship displayed a characteristic inward rectification, with no significant difference between the Ctrl and PD groups (*F*(6, 42.594) = 1.299, *p* = 0.278, repeated-measures linear mixed-effects model; Figure 2f, Table 3). Similarly, the input–output relationship, obtained by increasing the stimulation intensity from 0 to 50 μA, showed that the peak amplitude of evoked NMDA EPSCs increased with stimulus intensity and was comparable between groups (*F*(5, 64.401) = 1.510, *p* = 0.199, repeated-measures linear mixed-effects model; Figure 2g, Table 4). Subsequently, we compared AMPA-mediated EPSCs recorded at −70 mV with NMDA EPSCs recorded at +40 mV and calculated the AMPA/NMDA ratio in the presence of gabazine (10 μM). The AMPA/NMDA ratio was significantly higher in the PD condition. However, the absolute AMPAR- and NMDAR-mediated current amplitudes did not significantly differ between the Ctrl and PD conditions (PD: 2.08 ± 1.52, *n* = 9 vs. Ctrl: 1.01 ± 0.50, *n* = 8; *F*(1, 14.377) = 4.834, *p* = 0.045, Hedges’ *g* = 0.874, linear mixed-effects model; Figure 2h) (Supplementary Figure S1), indicating an altered relative contribution between AMPAR- and NMDAR-mediated synaptic transmission.

To additionally assess whether dopaminergic lesioning would affect inhibitory transmission in the M1 surface, we measured spontaneous inhibitory postsynaptic currents (sIPSCs) in layer 2 pyramidal neurons at a holding potential of 0 mV. This experiment was conducted with a Cs-based internal solution containing NBQX (10 μM) and AP5 (100 μM, an NMDAR antagonist) in order to eliminate excitatory synaptic inputs (Figure 2i). PD neurons exhibited pronounced decreases in both sIPSC amplitude and frequency compared to Ctrl neurons (PD_amplitude_: 25.49 ± 2.40, *n* = 5 vs. Ctrl_amplitude_: 29.50 ± 1.35, *n* = 7; *F*(1, 10) = 11.394, *p* = 0.007, Hedges’ *g* = −2.008, linear mixed-effects model; Figure 2j; PD_frequency_: 4.98 ± 1.40, *n* = 5 vs. Ctrl_frequency_: 10.70 ± 3.16, *n* = 7; *F*(1, 10) = 8.220, *p* = 0.017, Hedges’ *g* = −2.028, linear mixed-effects model; Figure 2k). This reduction in inhibitory synaptic activity indicates that dopaminergic degeneration reduced inhibitory synaptic transmission to the cortical surface neurons, which may further contribute to E-I imbalance within this M1 microcircuit, a common motif found in sensorimotor deficits [28,29].

### 2.3. Selective Impairment of mGluR_1/5_-Dependent LTD in PD

**Figure 3:**
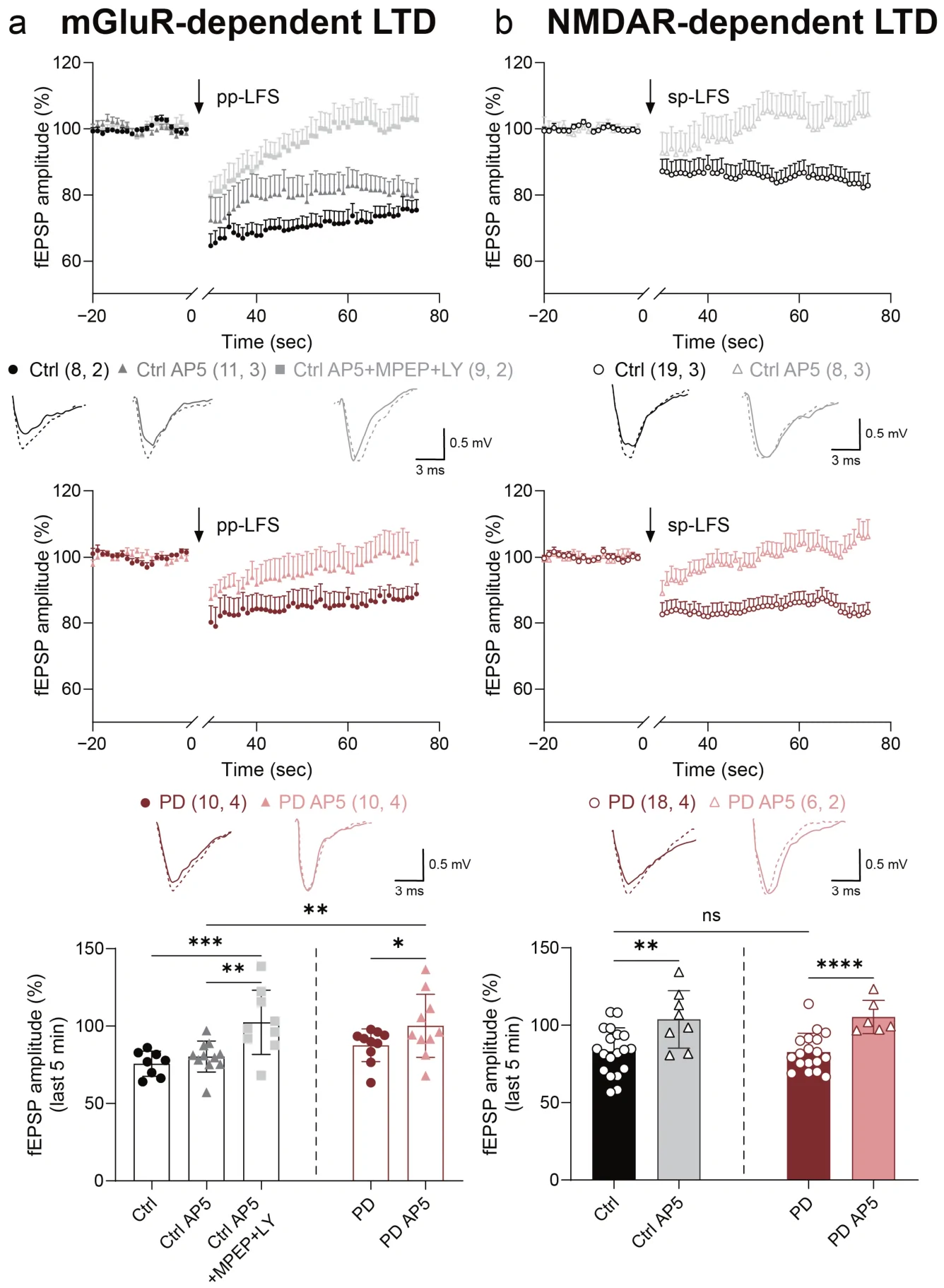
Induction of group I metabotropic glutamate receptor (mGluR_1/5_)- and N-methyl-D-aspartate receptor (NMDAR)-dependent long-term depression (LTD) on the M1 surface. (**a**) mGluR-dependent LTD induced by paired-pulse low-frequency stimulation (pp-LFS). Bath application of AP5 (100 μM, an NMDAR antagonist) did not affect LTD in control (Ctrl) slices, whereas co-application of 2-methyl-6-phenylethynyl-pyridine (MPEP, 10 μM; mGluR_5_ antagonist) and LY-367385 (100 μM; mGluR_1_ antagonist) completely abolished it, confirming the dependence of LTD on group I mGluRs. In Parkinson’s disease model (PD) slices, AP5 alone prevented LTD induction, indicating a selective loss of mGluR_1/5_-driven LTD. (**b**) NMDAR-dependent LTD induced by single-pulse LFS (sp-LFS) was comparable between Ctrl and PD slices. Gray indicates the Ctrl group, and red indicates the PD group. All data are presented as means ± standard deviation. * *p* < 0.05, ** *p* < 0.01, *** *p* < 0.005, **** *p* < 0.001.

To investigate the contribution of mGluRs to LTD on the M1 surface, we applied two well-established protocols for inducing LTD in cortical slices: (i) mGluR-dependent LTD (mGluR-LTD) induced by paired-pulse low-frequency stimulation (pp-LFS; 900 pairs of pulses delivered at 1 Hz with 50 ms inter-pulse intervals), and (ii) NMDAR-dependent LTD (NMDAR-LTD) induced by single-pulse low-frequency stimulation (sp-LFS; 900 single pulses at 1 Hz) [30,31,32]. We first verified that pp-LFS activated mGluRs but not NMDARs. Bath application of AP5 (100 μM, an NMDAR antagonist) had little effect on pp-LFS-induced LTD in the Ctrl M1 (Ctrl: 75.70% ± 8.08%, *n* = 8 vs. Ctrl_AP5_: 80.26% ± 10.01%, *n* = 11, *F*(1, 17) = 1.124, *p* = 0.304, linear mixed-effects model). However, the additional administration of 2-methyl-6-phenylethynyl-pyridine (MPEP, 10 μM), an mGluR_5_ antagonist, and (S)-(+)-α-amino-4-carboxy-2-methylbenzeneacetic acid (LY-367385; LY, 100 μM), an mGluR_1_ antagonist, completely eliminated LTD at 60 min post-induction, confirming that pp-LFS-induced LTD on the M1 surface is primarily driven by group I mGluRs (Ctrl: 75.70% ± 8.08%, *n* = 8 vs. Ctrl_AP5_: 80.26% ± 10.01%, *n* = 11 vs. Ctrl_AP5+MPEP+LY_: 102.46% ± 20.74%, *n* = 9; *F*(2, 25) = 9.305, *p* < 0.001, linear mixed-effects model). We next investigated whether PD affected mGluR-LTD on the M1 surface in the presence of AP5. Treatment with AP5 alone eliminated all LTD (PD: 87.60% ± 10.56%, *n* = 10 vs. PD_AP5_: 100.20% ± 20.34%, *n* = 10; *F*(1, 16.193) = 5.190, *p* = 0.037, linear mixed-effects model). Importantly, under AP5 treatment, mGluR-dependent LTD was significantly reduced in PD compared with Ctrl slices (PD_AP5_: 100.20% ± 20.34%, *n* = 10 vs. Ctrl_AP5_: 80.26% ± 10.01%, *n* = 11; *F*(1, 19) = 8.380, *p* = 0.009, linear mixed-effects model; Figure 3a).

To further examine whether PD also affected NMDAR-LTD, we applied sp-LFS to induce LTD in both Ctrl and PD slices, which was completely blocked by AP5 application in both groups (Ctrl: 83.12% ± 15.17%, *n* = 19 vs. Ctrl_AP5_: 103.80% ± 18.55%, *n* = 8; *F*(1, 25) = 9.179, *p* = 0.006, Hedges’ *g* = 1.239; PD: 82.69% ± 12.09%, *n* = 18 vs. PD_AP5_: 105.32% ± 10.76%, *n* = 6; *F*(1, 21.981) = 15.013, *p* < 0.001, Hedges’ *g* = 1.852, linear mixed-effects model). No significant difference in NMDAR-LTD amplitude was observed between the PD and Ctrl slices (PD: 82.69% ± 12.09%, *n* = 18 vs. Ctrl: 83.12% ± 15.17%, *n* = 19; *F*(1, 11.964) = 0.023, *p* = 0.882, Hedges’ *g* = −0.031, linear mixed-effects model). Therefore, PD appears to be specifically associated with changes in mGluR_1/5_-LTD at the M1 surface and does not appear to affect NMDAR-driven LTD (Figure 3b).

## 3. Discussion

**Figure 4:**
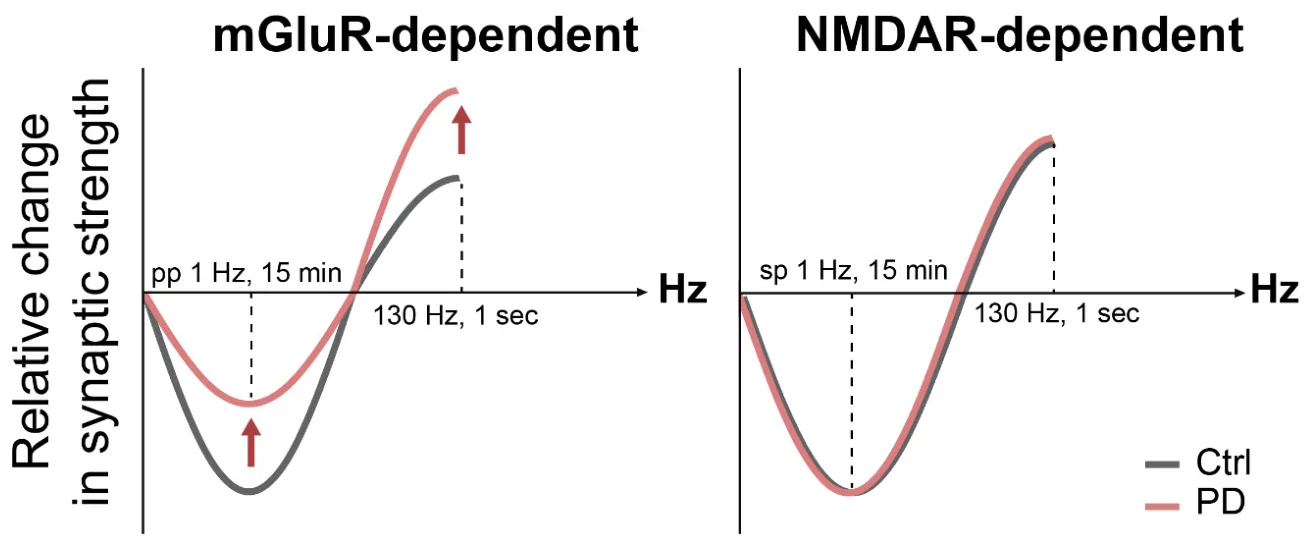
Schematic model of changes in group I metabotropic glutamate receptor (mGluR)-driven long-term synaptic plasticity in the superficial M1 of Parkinson’s disease model (PD) rats. mGluR_1/5_-mediated long-term potentiation was found to be significantly stronger in PD group than in control (Ctrl) group [11], whereas mGluR_1/5_-driven long-term depression is substantially weaker in PD group. NMDAR-dependent synaptic modulation is unaffected. Arrows indicate the direction of the change in relative synaptic strength in the PD condition compared with Ctrl.

Electrophysiological profiling of the M1 superficial layer revealed that intrinsic excitability, including the AP amplitude, AHP, threshold voltage, and membrane resistance, was preserved in the PD group; therefore, the increased cortical responsiveness did not appear to be attributable to changes in intrinsic membrane properties. Instead, the PD group exhibited significantly elevated AMPA/NMDA current ratios, without alterations in NMDAR current kinetics or input–output profiles, indicating an altered relative contribution of AMPAR- and NMDAR-mediated synaptic transmission. Additionally, sIPSC recordings showed marked reductions in both amplitude and frequency, reflecting weakened GABAergic inhibition and an excitatory–inhibitory imbalance. These findings collectively suggest that dopamine depletion is associated with an altered balance of excitatory glutamatergic transmission and reduced inhibitory tone, together with impaired mGluR_1/5_-mediated synaptic depression. Together with our previous report [11], these data suggest that dopaminergic degeneration leads to an overall shift in mGluR_1/5_-driven synaptic plasticity toward an increase in synaptic weight at the M1 surface (Figure 4).

Collectively, this excitatory shift may represent a compensatory form of motor homeostasis aimed at preserving the cortical output and motor learning in response to the loss of basal ganglia inputs to the M1 [33]. However, this compensation is likely to occur at the expense of synaptic flexibility, resulting in a reduced capacity for LTD and persistent cortical hyperexcitability.

Our findings further highlight the crucial role of mGluR_1/5_ signaling in the maintenance of cortical balance in PD, consistent with previous reports demonstrating close interactions between dopamine and mGluR-dependent LTD within the basal ganglia motor network [34]. Partial dopaminergic depletion has been shown to attenuate mGluR_1/5_-dependent plasticity via the indirect pathway [30,34,35,36], suggesting that dopamine loss causes a broad disruption of mGluR-mediated synaptic regulation across cortico-basal ganglia loops. However, the precise causal relationship between dopaminergic depletion and mGluR_1/5_-dependent LTD remains unclear, and to definitively address this question, a targeted loss-of-function study (for example, utilizing site-specific mGluR_1/5_ knockout mice) is required.

mGluR dysfunction is not unique to PD pathology, and it has been implicated in several other neurodegenerative and neuropsychiatric disorders including Huntington’s disease, Alzheimer’s disease, epilepsy, and depression [37,38,39,40,41,42,43]. Together, these findings identify altered excitatory–inhibitory balance and group I mGluR-dependent synaptic plasticity in the superficial M1 following dopaminergic depletion, and suggest that these mechanisms may represent potential targets for future investigation of cortical neuromodulation strategies (Table 5).

Several limitations in our study should be taken into consideration. Some experimental groups included a small number of animals, and all data were derived from male animals, which limit the clinical application of our findings to the broader human PD population. Nevertheless, the larger number of slices sampled from these animals yielded highly significant differences between groups, and strongly supported our findings. Sex differences in both 6-OHDA sensitivity [44] and PD motor symptoms [45] have been reported, and therefore M1 changes are also likely to exhibit sex-specific variation, which await further investigation. In addition, although the 6-OHDA model is a well-established tool for studying nigrostriatal dopaminergic degeneration, it does not recapitulate the progressive nature of PD or its other pathological characteristics, in particular synucleinopathy. As α-synuclein aggregates spread over time into the cortex and initiate a cascade of pathological changes, they are also likely to alter cortical M1 excitability and plasticity. Therefore, future studies may use additional models such as α-synuclein overexpression or pre-formed fibril injection to investigate alterations in mGluR_1/5_-driven M1 plasticity. Lastly, the observed electrophysiological changes have not been correlated with any behavioral changes, and thus the contribution of increased synaptic excitability and impaired mGluR-LTD to motor symptoms or learning remain unknown.

**Table 5:** Changes in metabotropic glutamate receptor (mGluR)-mediated long-term synaptic plasticity are associated with various neurodegenerative and neurological diseases. mGluR-driven long-term potentiation (LTP) or long-term depression (LTD) can be induced in various regions of the brain (induction methods shown in brackets) and suppressed or enhanced under disease conditions. ↓ indicates suppression, ↑ indicates enhancement of mGluR-LTP or mGluR-LTD in disease models. HFS, high-frequency stimulation; KO, knockout; LFS, low-frequency stimulation; MPEP, 2-methyl-6-phenylethynyl-pyridine; pp, paired-pulse; TBS, theta burst stimulation; DHPG, 3,5-dihydroxyphenylglycine; ACC, anterior cingulate cortex.

Disease	Animal	Recording Site	Synaptic Modulation	
			LTP	LTD
Parkinson’s disease [11] (This work)	Sprague-Dawley rat	Motor cortex superficial layer	↑ (HFS)	↓ (pp-LFS/LFS)
Hearing loss/tinnitus [7]	C57BL/6 mouse	Auditory cortex superficial layer	↓ or ↑ (Decreased pp-LFS-LTP/ increased TBS-LTP)	-
Huntington’s disease [46]	Wistar rat	Corticostriatal fibers	↑ (3-NP)	-
Alzheimer’s disease [47,48,49,50]	C57BL/6 mouse	Hippocampus CA3-CA1	↓ (HFS)	↑ or ↓ (DHPG/LFS)
Depression [51,52,53]	C57BL/6 mouse	Hippocampus CA3-CA1	-	↑ (pp-LFS/DHPG)
Epilepsy/seizure [54,55,56]	Wistar rat	Hippocampus CA3-CA1	↑ (TBS)	↓ (pp-LFS)
Fragile X syndrome [57,58,59,60,61]	*Fmr1* KO mouse	Hippocampus CA3-CA1	Affects LTP priming (DHPG + 100 Hz HFS)	↑ (pp-LFS/DHPG)
Chronic pain [62]	C57BL/6 mouse	ACC layer V-II/III, V-V/VI	-	↓ (DHPG + MPEP/LFS)

Overall, our findings propose several factors that may contribute to cortical surface hyperexcitability associated with nigral dopaminergic degeneration. Specifically, an altered relative contribution of AMPAR- and NMDAR-mediated transmission, together with reduced inhibitory tone, may contribute to disruption of the excitation–inhibition balance, while impaired mGluR_1/5_-dependent LTD may further favor network excitation. These cellular adaptations may represent a compensatory form of homeostasis, and pharmacological targeting of these pathways, in particular mGluR_1/5_, can be explored to improve motor function in PD.

## 4. Methods

### 4.1. Animals

Six-week-old male Sprague-Dawley rats were housed under a 12 h light/dark cycle in a temperature-controlled facility (25 °C) and allowed access to food and water ad libitum. All animal procedures were approved by the Institutional Animal Care and Use Committee at Incheon National University (Approval Code: INU-ANIM-2025-0034) and City University of Hong Kong (ANIMAL-2024-0101-A).

### 4.2. 6-OHDA-Induced Hemi-Parkinsonian Model in Rats

To generate the hemi-parkinsonian model in rats, dopaminergic neuron degeneration was induced via the stereotaxic administration of 6-OHDA (dissolved in normal saline with 0.02% ascorbic acid; Sigma-Aldrich, St. Louis, MO, USA) into the medial forebrain bundle. The animals were anesthetized by isoflurane inhalation (2%), and a burr hole was created in one hemisphere at −3.84 mm anterior and −1.4 mm lateral to the bregma. 6-OHDA was injected at a depth of 8.5 mm below the dura mater and an infusion rate of 0.5 µL/minute for 8 min, using a Hamilton syringe equipped with a micropump injector (26G needle). For electrophysiological experiments, recordings from the hemisphere ipsilateral to the 6-OHDA lesion were defined as the PD condition, and those from the contralateral hemisphere of the same animal as the contralateral Ctrl condition. The contralateral hemisphere was used as an internal control.

All animals were first subjected to apomorphine-induced rotation testing to confirm successful establishment of the unilateral 6-OHDA lesion model. Only animals exhibiting at least three net contralateral rotations per minute were included in subsequent experiments. After behavioral validation, animals were randomly assigned to either histological or electrophysiological experimental endpoints. Because histological analysis required transcardial perfusion and tissue fixation, the same animals could not be used for electrophysiological recordings. Therefore, TH immunohistochemistry and electrophysiological recordings were performed in separate cohorts derived from the same behaviorally validated population. TH depletion was confirmed in the histological cohort but not in the electrophysiological cohort.

### 4.3. Apomorphine-Induced Rotation Test

To screen for 6-OHDA-induced dopamine depletion, the rats were administered apomorphine (0.5 mg/kg in 0.1% ascorbic acid, subcutaneous; Biosynth, Compton, Berkshire, UK) and placed individually in a 30 cm-diameter cylinder. Rotating behavior was recorded using a digital video camera. The number of rotations during a 30 min observation period was manually counted for each rat. The net number of rotations was calculated as the number of contralateral rotations (away from the lesioned side) minus the number of ipsilateral rotations. PD symptoms were defined as the exhibition of at least three net contralateral rotations per minute.

### 4.4. Rotarod Test

The rats were trained to walk on a rotarod apparatus (Ugo Basile, Gemonio, VA, Italy) prior to testing. Fourteen days after 6-OHDA administration, the rats were subjected to the rotarod test at a rotation speed of 10 rpm for at least 180 s. The speed was gradually increased at a rate of 1 rpm every 6 s, and the latency to fall from the rod was recorded in two trials per rat. Data are presented as the ratio of the latency to fall after 6-OHDA administration to that before 6-OHDA administration (%).

### 4.5. Immunohistochemistry

Rats were deeply anesthetized via isoflurane inhalation (2%) and perfused transcardially with phosphate-buffered saline (PBS) and 4% paraformaldehyde (PFA). The brains were dissected and submerged in 4% PFA for 24 h at 4 °C, then submerged in 30% sucrose. The brains were then cut into 50 µm-thick coronal frozen sections using a cryostat (CM1520; Leica Biosystems, Nussloch, Germany). For TH staining, free-floating sections were washed with 0.3% Triton X-100 in PBS for 30 min, then incubated overnight with a rabbit anti-TH antibody (1:1000 dilution, AB152, RRID: AB_390204; Millipore, Burlington, MA, USA). After washing with PBS, the sections were incubated with a biotinylated secondary anti-rabbit antibody (1:200 dilution, MP-7401, RRID: AB_2336529; Vector Labs, Newark, CA, USA) for 1 h. The sections were incubated with 3,3-diaminobenzidine for 3–5 min to visualize antibody labeling and mounted on aminosilane-coated slides. The area of TH-positive staining in the ipsilateral hemisphere was normalized to that in the contralateral hemisphere (100%).

### 4.6. Brain Slice Preparation

Primary motor cortical slices were collected from rats with hemi-parkinsonism. The animals were deeply anesthetized with 2% isoflurane, and the brains were rapidly removed into chilled, oxygenated dissection buffer. Slices for whole-cell patch-clamp recording were prepared in a choline-based slicing buffer containing 20.0 mM glucose, 110.0 mM choline chloride, 2.5 mM KCl, 1.2 mM NaH_2_PO_4_, 7.0 mM MgCl_2_, and 0.5 mM CaCl_2_. Slices for extracellular field recording were prepared in a sucrose-based slicing buffer containing 75.0 mM sucrose, 25.0 mM glucose, 87.0 mM NaCl, 2.5 mM KCl, 1.3 mM NaH_2_PO_4_, 25.0 mM NaHCO_3_, 7.0 mM MgCl_2_, and 0.5 mM CaCl_2_.

Coronal M1 slices were cut using a vibratome (VT1200S; Leica, Nussloch, Germany) at a thickness of 400 μm for field recordings and 300 μm for patch-clamp recordings. The cut slices were transferred to artificial cerebrospinal fluid (ACSF; 20.0 mM glucose, 124.0 mM NaCl, 2.5 mM KCl, 1.0 mM NaH_2_PO_4_, 26.2 mM NaHCO_3_, 1.3 mM MgCl_2_, and 2.5 mM CaCl_2_ for patch recordings; 25.0 mM glucose, 125.0 mM NaCl, 2.5 mM KCl, 1.3 mM NaH_2_PO_4_, 25.0 mM NaHCO_3_, 1.0 mM MgCl_2_, and 2.0 mM CaCl_2_ for field recordings), which was continuously bubbled with 95% O_2_/5% CO_2_. All slices were incubated at 32 °C for 12 min, allowed to recover for 1 h at room temperature, then transferred to a submersion-type recording chamber perfused with oxygenated ACSF at 31–32 °C.

### 4.7. Whole-Cell Patch-Clamp Recording

Whole-cell recordings of layer 2 pyramidal neurons in the M1 were performed. Recording pipettes (3–7 MΩ) were pulled from borosilicate glass and filled with different solutions depending on the experiment. To evaluate intrinsic membrane properties, the pipettes were filled with a potassium-based internal solution containing (in mM) 135 K-gluconate, 7 NaCl, 10 HEPES, 0.2 EGTA, 4 Na_2_ATP, and 0.2 NaGTP (pH 7.3, adjusted with KOH; 292–293 mOsm). For spontaneous inhibitory postsynaptic current (sIPSC) recordings, the pipettes were filled with a cesium-based internal solution containing (in mM) 135 CsMeSO_3_, 10 CsCl, 10 HEPES, 0.2 EGTA, 4 Na_2_ATP, 0.4 NaGTP, and 5 QX-314 (pH 7.3, adjusted with CsOH; 292–293 mOsm). RMP was measured 3 min after break-in. Intrinsic excitability was assessed in current-clamp mode by biasing the membrane potential to −70 mV via steady current injection and delivering step currents from −400 to +800 pA in 100-pA increments. R_in_ was determined from the voltage responses to a current injection of −100 pA. AP properties were quantified using the first spike elicited by the minimal suprathreshold-depolarizing current injection. AP amplitude was calculated as the voltage difference between the AP threshold and the peak membrane potential. AHP amplitude was measured as the difference between the threshold voltage and the most negative membrane potential reached after AP. AP threshold was defined as the membrane potential at which the first derivative of voltage over time (dV/dt) exceeded 20 mV/ms. To pharmacologically isolate inhibitory events, sIPSCs were recorded at 0 mV using the Cs-based internal solution in the presence of NBQX (Tocris, Bristol, UK, 10 μM) and AP5 (Sigma-Aldrich, St. Louis, MO, USA, 100 μM). sIPSCs were analyzed from 3 min recording epochs. For each cell, a stable 1 min segment of continuous recording was selected for analysis. Signals were Gaussian-filtered at 300 Hz, and events were detected using a fixed amplitude threshold of 20 pA. Event detection and analysis were performed blinded to experimental condition.

Pyramidal neurons were identified under infrared differential interference contrast microscopy based on their characteristic pyramidal-shaped soma and prominent apical dendrites, and were further confirmed by regular-spiking firing patterns. Cells were included for analysis only if they exhibited a stable resting membrane potential (<−60 mV), and an input resistance within the typical range for cortical pyramidal neurons (50–150 MΩ). Access resistance was monitored throughout the recording and was typically 20–30 MΩ. Recordings were excluded when the input resistance changed by more than 15% from the baseline value. For long-term plasticity experiments, stable baseline responses were recorded for at least 20 min before LTD induction.

### 4.8. Evoked Patch-Clamp Recording

Evoked synaptic responses of superficial layer pyramidal neurons in the M1 were recorded and used to measure the NMDAR-mediated currents and AMPA/NMDA ratios. Recordings were performed in voltage-clamp mode using pipettes (3–7 MΩ) filled with a cesium-based internal solution as described above. Slices were continuously perfused with oxygenated ACSF at 32 °C. EPSCs were evoked using a concentric bipolar electrode (FHC, Bowdoin, ME, USA), through which electrical stimuli (0.1 Hz, 100-μs pulse width) were delivered.

Neurons were voltage-clamped at +40 mV in the presence of NBQX (10 μM; Tocris, Bristol, UK) and gabazine (10 μM; Sigma-Aldrich, St. Louis, MO, USA). Treatment with AP5 (100 µM; Sigma-Aldrich, St. Louis, MO, USA) completely abolished the remaining current, confirming the pharmacological isolation of NMDAR-mediated responses. The voltage dependence of NMDAR currents was assessed across holding potentials from +40 to −80 mV, and input–output relationships were generated by increasing the stimulation intensity from 0 to 50 μA in 10-μA increments. AMPA-mediated EPSCs were recorded at −70 mV. NMDAR-mediated EPSCs at +40 mV were quantified using a time-window-based approach. The NMDAR component was measured at 50 ms after stimulus, when the fast AMPAR-mediated current had decayed to baseline, allowing isolation of the slow NMDAR-mediated component. AMPAR-mediated EPSCs were measured at −70 mV at peak amplitude. The AMPA/NMDA ratio was calculated as the peak current at −70 mV divided by the NMDA current measured at +40 mV.

### 4.9. Extracellular Field Recording

Field excitatory postsynaptic potentials (fEPSPs) were recorded from the superficial layer of the M1 using ACSF-filled glass microelectrodes. Synaptic responses were evoked by single-pulse stimulation delivered through a concentric bipolar microelectrode (FHC) positioned in the same superficial layer. The distance between the stimulating and recording electrodes was maintained at 300–500 µm (typically 400 µm) across all slices. To standardize the baseline responses across slices, the stimulation intensity was first determined by constructing an input–output curve and then adjusted to evoke an fEPSP amplitude corresponding to approximately 50% of the maximal response. Once the intensity was set, fEPSPs were recorded until a stable baseline was obtained for at least 20 min prior to LTD induction. LTD was induced using either pp-LFS (900 paired pulses at 1 Hz with a 50 ms inter-pulse interval) or sp-LFS (900 single pulses at 1 Hz), depending on the experimental condition. Following LFS, fEPSPs were continuously monitored for at least 60 min, and the synaptic strength was quantified by normalizing the fEPSP amplitudes to the pre-induction baseline. LTD magnitude was calculated as the average normalized fEPSP amplitude during the final 5 min of the recording period.

For pharmacological experiments, AP5 (100 µM; Sigma-Aldrich, St. Louis, MO, USA), MPEP (10 μM; Tocris, Bristol, UK), and LY-367385 (100 μM; Sigma-Aldrich, St. Louis, MO, USA) were applied via continuous bath perfusion through the recording chamber. Drug perfusion was initiated after baseline stabilization and maintained throughout the recording period (including the LTD induction and post-induction phases) to ensure consistent drug exposure during both baseline monitoring and synaptic plasticity expression.

### 4.10. Statistics

All electrophysiology data were acquired using a MultiClamp 700B amplifier, digitized using a Digidata 1550B system, and analyzed offline using pCLAMP 11.4 software (Molecular Devices, San Jose, CA, USA). All statistical analyses were performed using SPSS 28.0 (IBM Corp., Armonk, NY, USA). For comparisons between two groups, two-sided independent-sample *t*-tests or linear mixed-effects models were used. All repeated-measures analyses were performed using linear mixed-effects models and report the interaction term between the experimental condition and the repeated factor. Statistical significance was set at *p* < 0.05. Hedges’ *g* or η^2^ were determined to estimate effect sizes. All graphs were generated using GraphPad Prism 8 (GraphPad Software Inc., La Jolla, CA, USA), and the final arrangements and labeling were completed using Adobe Illustrator CC 2019 (Adobe Inc., San Jose, CA, USA). All data are presented as means ± standard deviation, and normalization for comparison is noted where relevant.

## Figures and Tables

**Table 1:** Statistical details related to Figure 2b. All data are presented as means ± standard deviation. Statistical significance was determined using a repeated-measures linear mixed-effects model. *n* denotes the number of slices and *N* denotes the number of animals. Ctrl, contralateral hemisphere; PD, ipsilateral 6-OHDA-lesioned hemisphere from the same hemi-parkinsonian animals.

Group (*n*, *N*)	Stimulus Intensity (µA)								Repeated-Measures Linear Mixed-Effects Model
	50	100	150	200	250	300	350	400	
Ctrl (28, 10)	0.35 ± 0.15	0.79 ± 0.28	0.99 ± 0.30	1.11 ± 0.31	1.22 ± 0.33	1.31 ± 0.32	1.39 ± 0.32	1.46 ± 0.34	*F*(7, 392.994) = 2.029, *p* = 0.049
PD (31, 12)	0.43 ± 0.29	0.91 ± 0.38	1.17 ± 1.17	1.35 ± 0.43	1.50 ± 0.47	1.61 ± 0.50	1.72 ± 0.54	1.78 ± 0.57	
Hedges’ *g*	0.34	0.35	0.2	0.63	0.67	0.7	0.73	0.67	

**Table 2:** Statistical details related to Figure 2c. All data are presented as means ± standard deviation. Statistical significance was determined using a repeated-measures linear mixed-effects model. *n* denotes the number of slices and *N* denotes the number of animals. Ctrl, contralateral hemisphere; PD, ipsilateral 6-OHDA-lesioned hemisphere from the same hemi-parkinsonian animals.

Group (*n*, *N*)	Current Injection (pA)									Partial η^2^
	0	100	200	300	400	500	600	700	800	
Ctrl (11, 2)	0.00 ± 0.00	0.00 ± 0.00	0.18 ± 0.60	3.09 ± 2.66	6.27 ± 5.06	9.36 ± 6.95	12.55 ± 8.73	15.72 ± 9.06	18.27 ± 9.65	0
PD (10, 2)	0.00 ± 0.00	0.00 ± 0.00	0.30 ± 0.95	2.20 ± 2.86	6.70 ± 4.22	10.50 ± 4.81	14.50 ± 5.13	17.70 ± 5.33	20.50 ± 5.99	

**Table 3:** Statistical details related to Figure 2f. All data are presented as means ± standard deviation. Statistical significance was determined using a repeated-measures linear mixed-effects model. *n* denotes the number of slices and *N* denotes the number of animals. Ctrl, contralateral hemisphere; PD, ipsilateral 6-OHDA-lesioned hemisphere from the same hemi-parkinsonian animals.

	Membrane Potential (mV)							
Group (*n*, *N*)	40	20	0	−20	−40	−60	−80	Partial η^2^
Ctrl (5, 2)	1.00 ± 0.00	0.37 ± 0.21	−0.15 ± 0.22	−0.59 ± 0.46	−1.07 ± 0.64	−1.75 ± 0.79	−1.23 ± 0.95	0.098
PD (5, 2)	1.00 ± 0.00	0.55 ± 0.22	−0.12 ± 0.53	−0.69 ± 0.82	−1.33 ± 1.07	−1.31 ± 0.63	−0.49 ± 0.14	

**Table 4:** Statistical details related to Figure 2g. All data are presented as means ± standard deviation. Statistical significance was determined using a repeated-measures linear mixed-effects model. *n* denotes the number of slices and *N* denotes the number of animals. Ctrl, contralateral hemisphere; PD, ipsilateral 6-OHDA-lesioned hemisphere from the same hemi-parkinsonian animals.

	Intensity (μA)						
Group (*n*, *N*)	0	10	20	30	40	50	Partial η^2^
Ctrl (7, 2)	0.08 ± 0.05	0.62 ± 0.43	1.03 ± 0.67	1.20 ± 0.78	1.26 ± 0.87	1.27 ± 0.93	0.012
PD (8, 2)	0.06 ± 0.05	0.38 ± 0.12	1.01 ± 0.48	1.26 ± 0.57	1.32 ± 0.68	1.38 ± 0.74	

## Data Availability

All data generated or analyzed during this study are included in this published article.

## Supplementary Materials

The following supporting information can be downloaded at https://www.mdpi.com/article/10.3390/ijms27177564/s1.
