# Supplementary material for: Altered Excitation–Inhibition Balance and mGluR1/5-Driven Plasticity in the Motor Cortical Surface in a Rat Model of Parkinson’s Disease
Source: Int J Mol Sci. 2026 Aug 24;27(17):7564. doi: 10.3390/ijms27177564 (PMC13565811; doi:10.3390/ijms27177564)
Supplement: Supplementary file 1 [file ijms-27-07564-s001.zip › ijms-4476310-supplementary.pdf]

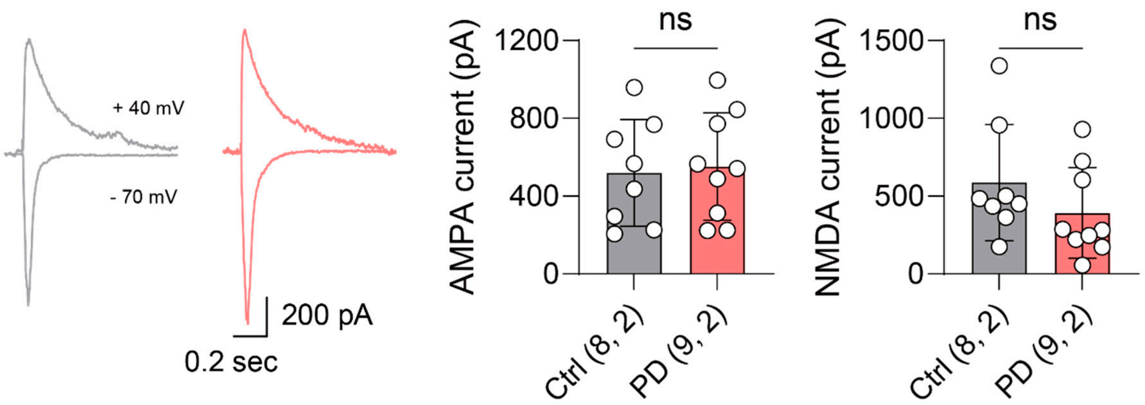

Supplementary Figure S1. Absolute AMPAR- and NMDAR-mediated synaptic currents in superficial M1 pyramidal neurons. Representative traces of AMPAR- and NMDAR-mediated excitatory postsynaptic currents (EPSCs) recorded from superficial-layer M1 pyramidal neurons in the contralateral control (Ctrl) and Parkinson's disease model (PD) hemispheres. AMPAR-mediated EPSCs were recorded at -70 mV, whereas NMDAR-mediated EPSCs were recorded at +40 mV. Summary plots show the absolute peak amplitudes of AMPAR-mediated currents and NMDAR-mediated currents. No significant differences were observed between the Ctrl and PD conditions for either AMPAR- or NMDAR-mediated current amplitudes. All data are presented as means ± standard deviation. ns; not significant.
